# Hsa_circ_0018818 knockdown suppresses tumorigenesis in non-small cell lung cancer by sponging miR-767-3p

**DOI:** 10.18632/aging.103089

**Published:** 2020-05-01

**Authors:** Xiaohui Xu, Xiaoyun Zhou, Chao Gao, Yushang Cui

**Affiliations:** 1 Department of Thoracic Surgery, Peking Union Medical College Hospital, Beijing 100730, China; 2 Peking Union Medical College, Chinese Academy of Medical Sciences, Beijing 100730, China

**Keywords:** hsa_circ_0018818, NSCLC, miR-767-3p, NID1

## Abstract

To identify potential therapeutic targets in non-small cell lung cancer NSCLC, we conducted a bioinformatics analysis of circRNAs differentially expressed between NSCLC tissues and adjacent normal tissues. Cell proliferation and apoptosis was assessed using CCK-8 and flow cytometry, respectively. A connection between hsa_circ_0018818 and miR-767-3p was confirmed in dual luciferase reporter assays. Gene and protein expression in NSCLC cells were measured using quantitative PCR and Western-blotting, respectively. And a xenograft tumor model was established to assess the function of hsa_circ_0018818 in NSCLC *in vivo*. Hsa_circ_0018818 was greatly upregulated in NSCLC tumor tissues. Knocking down hsa_circ_0018818 using a targeted shRNA inhibited the proliferation and invasiveness of NSCLC cells and induced their apoptosis via the miR-767-3p/Nidogen 1 (NID1) signaling axis. Hsa_circ_0018818 knockdown also inactivated Epithelial-mesenchymal transition (EMT) process and PI3K/Akt signaling. In summary, hsa_circ_0018818 knockdown inhibited NSCLC tumorigenesis *in vitro* and *in vivo*, which suggests it could potentially serve as a target for the treatment of NSCLC.

## INTRODUCTION

Lung cancers are the most commonly diagnosed malignant tumors and a major cause of cancer-related death throughout the world [1]. Among those, non-small cell lung cancers (NSCLC) make up approximately 83% of all lung cancers. Moreover, about 80% of patients with NSCLC are diagnosed at advanced stages [2]. Although much effort has been made to improve treatment of NSCLC, the prognosis remains poor [3]. It is therefore urgent to find a new biomarker for diagnosis of NSCLC at earlier stages, which could potentially improve prognoses and outcomes among patients with NSCLC.

Circular RNA (circRNA) is an endogenous RNA with a covalently closed cyclic structure [4]. Intracellular circRNAs with competing endogenous RNA (ceRNA) activity may function as microRNA (miRNA) antagonists by binding to microRNA recognition elements (MREs) on target genes. This would suppress the activity of miRNA, thereby enhancing expression of target genes [5, 6]. For that reason, circRNAs are considered important biological regulators when exploring the molecular mechanisms of diseases or seeking to identify therapeutic markers. For example, recent studies have shown the importance of circRNAs in modulating cancer-related signaling pathways [7, 8]. In addition, particular circRNAs may be related to specific types of malignant tumors and serve as key factors for tumorigenesis [9–11]. However, the function of circRNAs during the progression of NSCLC remains largely unexplored. Therefore, in an effort to identify potential targets for the development of novel therapeutic strategies against NSCLC, we used systemic bioinformatics analysis to identify circRNAs essential for the biological processes of NSCLC.

## RESULTS

### Differentially expressed circRNAs in NSCLC

**Figure 1:**
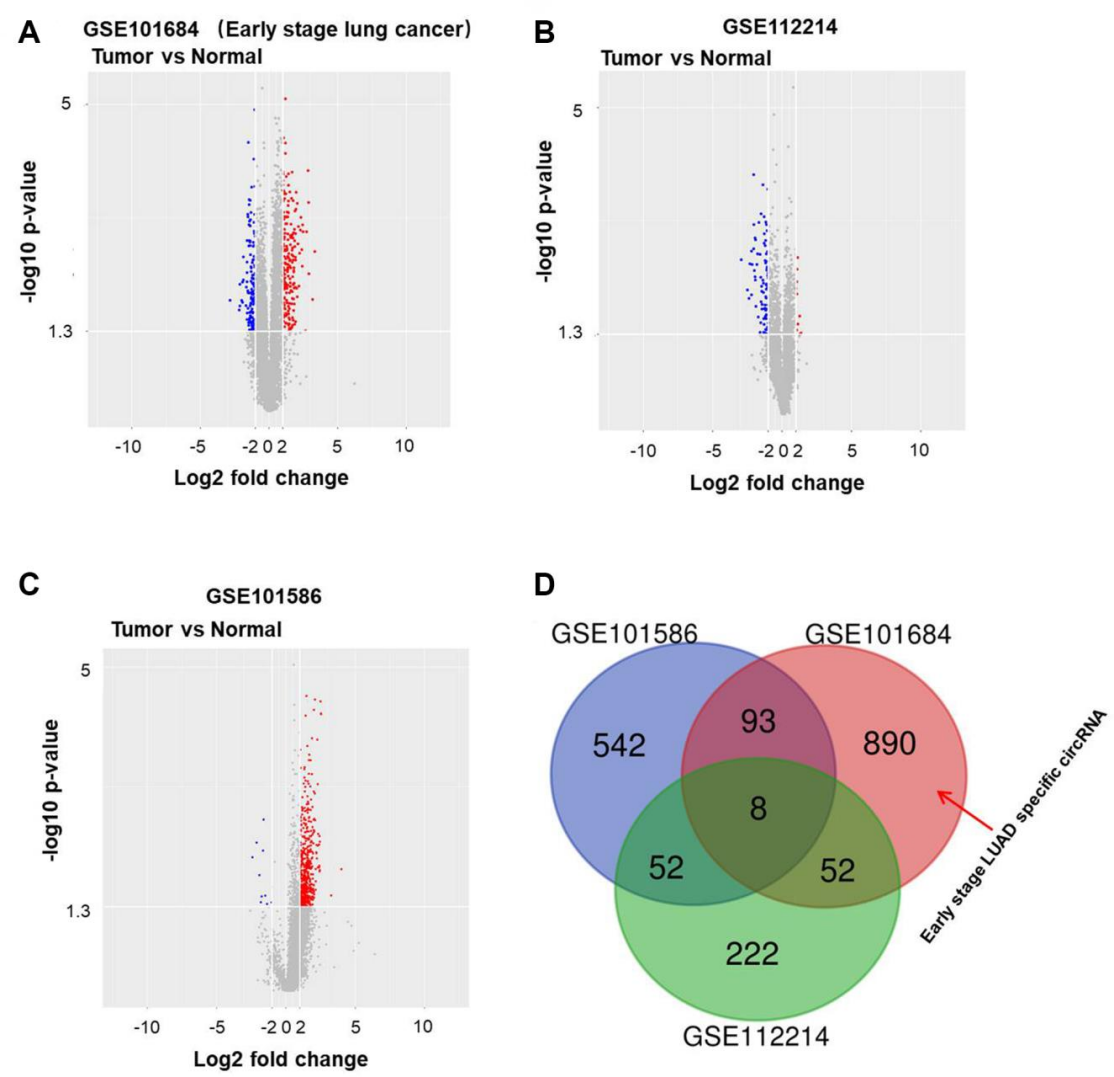
**Differentially expressed circRNAs in NSCLC.** Volcano plots illustrating the circRNAs differentially expressed in NSCLC detected in the (**A**) GSE101684, (**B**) GSE112214 and (**C**) GSE101586 datasets. Red indicates a higher expression level, while blue indicates a lower expression level. (**D**) Venn diagram showing the overlap among the differentially expressed circRNAs in the three datasets. In GSE101684, 890 circRNAs were related to NSCLC.

To detect differentially expressed circRNAs in NSCLC, we performed a bioinformatics analysis. In Figure 1A–1C, circRNAs differentially expressed as compared to normal tissues in the GSE101586, GSE101586 and GSE112214 datasets are presented as volcano plots. Overlap among the three datasets is illustrated by the Venn diagram in Figure 1D. Among the differentially expressed circRNAs, 890 in GSE101684 were related to early stage NSCLC.

**Figure 2:**
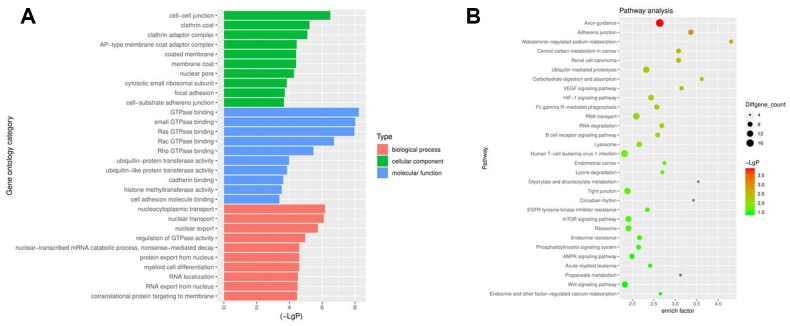
**Profiles of circRNAs in NSCLC analyzed with GO and pathway analyses.** (**A**) Go analysis exploring the potential functions of differentially expressed circRNAs. (**B**) Pathway analysis exploring the signaling pathways related to NSCLC.

Gene ontology (Go) and pathway analyses performed to determine the host genes of the circRNAs showed that the most common biological process was nucleocytoplasmic transport. The most enriched cellular component was cell-cell junction, and the most enriched molecular function was GTPase binding (Figure 2A). Pathway analysis revealed that the phosphatidylinositol 3 kinase (PI3K) signaling pathway was related to the development of NSCLC (Figure 2B).

### Silencing hsa_circ_0018818 significantly inhibits NSCLC cell proliferation

**Figure 3:**
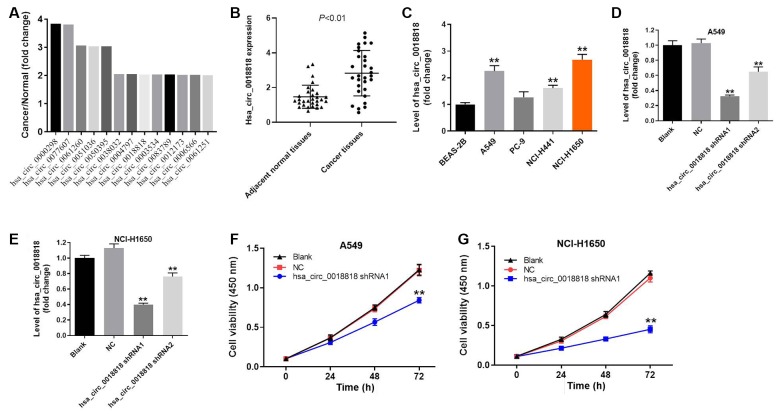
**Downregulation of hsa_circ_0018818 inhibits NSCLC cells proliferation.** (**A**) The overlap of these differentially expressed circRNAs was analyzed. (**B**) RT-qPCR analysis of hsa_circ_0018818 gene expression in NSCLC and adjacent normal tissues. (**C**) RT-qPCR analysis of hsa_circ_0018818 gene expression in BEAS-2B, A549, PC-9, NCI-H1441 and NCI-H1650 cells. (**D**, **E**) Suppression of hsa_circ_0018818 expression after transfection of A549 and NCI-H1650 cells with shRNA for 24 h. (**F**, **G**) CCK-8 assays of A549 and NCI-H1650 cell proliferation. Shown are the OD_450_ values. **P< 0.01 vs. control.

The top differentially expressed circRNAs in GSE101684 were related to early stage NSCLC (Figure 3A). Among them, hsa_circ_0018818 (host gene: USP54) closely correlated with NSCLC tumorigenesis. Therefore, hsa_circ_0018818 was selected for further analysis. In addition, hsa_circ_0018818 was significantly upregulated in NSCLC tissues compared with adjacent normal tissues (Figure 3B). Besides, hsa_circ_0018818 was closely associated with the metastasis of NSLC (Table 1). RT-qPCR analysis showed that hsa_circ_0018818 expression was upregulated in several NSCLC cell lines as compared to BEAS-2B normal lung epithelial cells (Figure 3C). Further analysis of A549 and NCI-H1650 cells showed that transfecting the cells with shRNA1 or shRNA2 targeting hsa_circ_0018818 significantly downregulated its expression (Figure 3D, 3E). Although both shRNAs were stably transfected, hsa_circ_0018818 shRNA1 exhibited better transfection efficiency and was therefore used in subsequent experiments. CCK-8 assays demonstrated that silencing of hsa_circ_000018818 significantly inhibited NSCLC cell proliferation (Figure 3F, 3G).

**Table 1:** The correlation of hsa_circ_0018818 and clinic-pathological parameters of patients with NSCLC.

**Parameters**	**No. of patients**	**Mean ± SD**	***p* value**
Age			0.619
≤ 50	18	2.711 ± 1.208	
> 50	12	2.061 ± 0.828	
Smoking			0.656
Yes	17	2.608 ± 1.070	
No	13	2.133 ± 0.928	
Tumor volume			
≤ 3 cm	13	2.501 ± 1.109	0.571
> 3 cm	17	2.266 ± 0.899	
Lymph node metastasis			0.008**
N0-N1	18	3.768 ± 1.322	
N2-N3	12	1.461 ± 0.887	
Distant metastasis			0.058*
M0	17	3.432 ± 1.322	
M1	13	1.885 ± 0.821	
TNM stage			0.389
I-II	15	2.934 ± 1.181	
III- IV	15	2.115 ± 0.923	

*P<0.05, **P<0.01, student’s t test.

TNM: Tumor Node Metastasis.

### Hsa_circ_0018818 shRNA1 induces apoptosis and reduces the invasiveness of NSCLC cells

**Figure 4:**
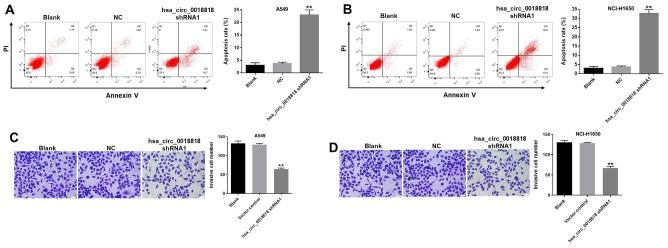
**Hsa_circ_0018818 shRNA1 induces apoptosis and inhibits invasion by NSCLC cells.** (**A**, **B**) The incidence of apoptosis was detected using FACS after double staining cells with Annexin V and PI. X axis: the level of Annexin-V FITC fluorescence; Y axis: the PI fluorescence. (**C**, **D**) Transwell assays testing the invasiveness of A549 and NCI-H1650 cells. Magnification: 400×. **P< 0.01 compared to control.

Flow cytometry exemplified by the results presented in Figure 4A, 4B showed that downregulating hsa_circ_0018818 obviously induced apoptosis among both A549 and NCI-H1650 cells. Moreover, transwell assays revealed that transfection with hsa_circ_0018818 shRNA1 substantially reduced the invasiveness of these cells (Figure 4C, 4D). Because, NCI-H1650 cells were more sensitive to hsa_circ_0018818 shRNA1 than A549 cells, NCI-H1650 cells were used in the following experiments.

### MiR-767-3p is a downstream target of hsa_circ_0018818

**Figure 5:**
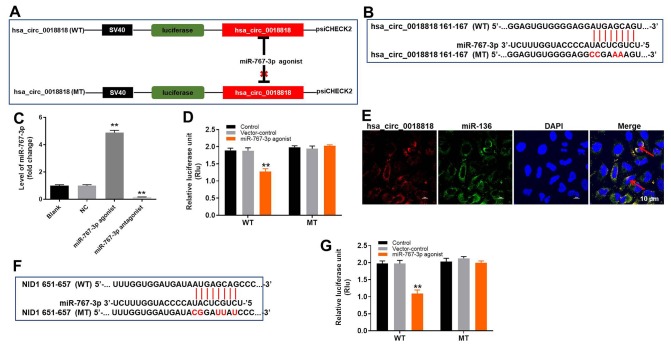
**MiR-767-3p is the downstream target of hsa_circ_0018818.** (**A**, **B**) Gene structure of hsa_circ_0018818 indicating the predicted miR-767-3p binding site in its 3'UTR. (**C**) RT-qPCR analysis miR-767-3p expression in NCI-H1650 cells. (**D**) The luciferase activity in NCI-H1650 cells after co-transfecting a plasmid encoding the wild-type (WT) or mutant (MT) hsa_circ_0018818 3′-UTR and miR-767-3p. (**E**) Co-localization of hsa_circ_0018818 and miR-767-3p detected using FISH. **P< 0.01 vs. control. (**F**) Gene structure of NID1 at the position of bp 161-167 showing the predicted miR-767-3p binding site in its 3'UTR. (**G**) Luciferase activity in NCI-H1650 cells after co-transfecting a plasmid encoding the WT or MT NID1 3′-UTR and miR-767-3p. **P< 0.01 vs. control.

To investigate the mechanism by which hsa_circ_0018818 regulates the progression of NSCLC, its interactome was examined using the web tool CircInteractome (https://circinteractome.nia.nih.gov/). We found that miR-767-3p was the most likely downstream target of hsa_circ_0018818 (Figure 5A, 5B). In addition, RT-qPCR analysis demonstrated that miR-767-3p expression was notably upregulated by miR-767-3p agonist and but downregulated by miR-767-3p antagonist (Figure 5C). Dual luciferase reporter assays confirmed that miR-767-3p is a downstream target of hsa_circ_0018818 (Figure 5D). This was further verified by fluorescence in situ hybridization (FISH), which showed their colocalization with cells (Figure 5E). Taken together, these findings indicate that miR-767-3p is a downstream target of hsa_circ_0018818.

### Nidogen 1 (NID1) is a direct target of miR-767-3p

To determine the target of miR-767-3p, Targetscan (http://www.targetscan.org/vert_71/), miRDB (http://www.mirdb.org/), and dual luciferase assays were used. As illustrated in Figure 5F, 5G, NID1 is a direct target of miR-767-3p.

### Hsa_circ_0018818 knockdown inhibits NSCLC progression by inactivating PI3K signaling

**Figure 6:**
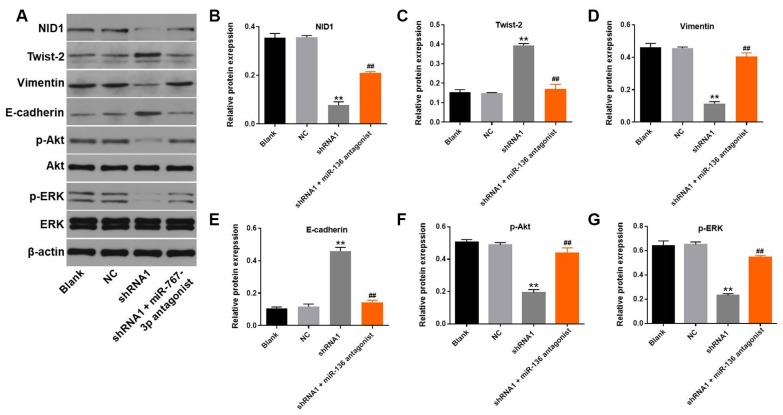
**Silencing Hsa_circ_0018818 inhibits NSCLC progression by inactivating EMT process and PI3K/Akt signaling.** (**A**) Western blot analysis of NID1, E-cadherin, Vimentin, Twist-2, Akt, ERK, p-Akt and p-ERK expression in NCI-H1650 cells. (**B**–**G**) Relative levels of NID1, Vimentin, E-cadherin, Twist-2,p-Akt and p-ERK expression in NCI-H1650 cells normalized to β-actin expression. **P< 0.01 vs. control. ^##^P< 0.01 vs. shRNA1.

Subsequent western blot analysis demonstrated that hsa_circ_0018818 knockdown significantly decreased expression of NID1 (Figure 6A, 6B). This inhibitory effect of hsa_circ_0018818 shRNA1 on NID1 was partially reversed by miR-767-3p antagonist (Figure 6B). Moreover, expression of Twist-2 and E-cadherin in NSCLC cells was notably increased by knockdown of hsa_circ_0018818. In contrast, hsa_circ_0018818 shRNA1 greatly decreased the expression of Vimentin. Meanwhile, downregulation of miR-136 partially suppressed the inhibitory effect of hsa_circ_0018818 shRNA on EMT process of NSCLC (Figure 6A, 6C–6E). Besides, expression of p-Akt and p-ERK in NSCLC cells was significantly downregulated by hsa_circ_0018818 knockdown, but was partially rescued in the presence of miR-767-3p antagonist (Figure 6A, 6F, 6G). This suggests that hsa_circ_0018818 silencing inhibits the progression of NSCLC by inactivating EMT process and PI3K/Akt signaling.

### Akt inhibitor further enhanced the inhibitory effect of hsa_circ_0018818 shRNA on the progression of NSCLC

**Figure 7:**
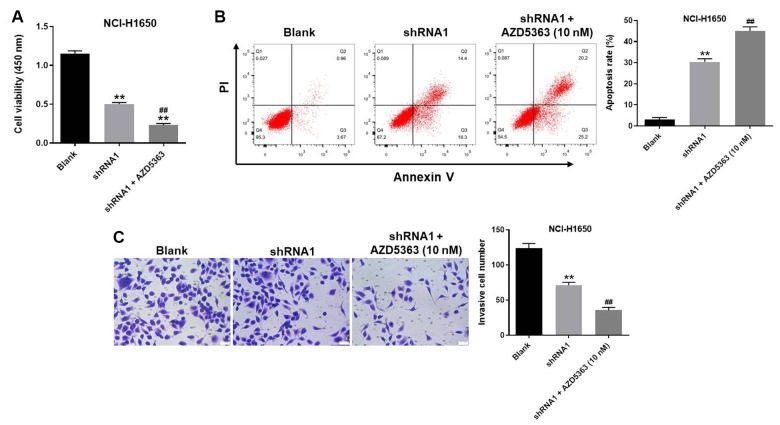
**Akt inhibitor further enhanced the inhibitory effect of hsa_circ_0018818 shRNA on progression of NSCLC.** (**A**) The OD value of NCI-H1650 cells was detected by CCK-8 assay. (**B**) The apoptotic NSCLC cells were examined by flow cytometry. (**C**) The invasion of NSCLC cells was tested by transwell assay. **P< 0.01 vs. control. ^##^P< 0.01 vs. shRNA1.

To further verify the mechanism by which hsa_circ_0018818 mediated the progression of NSCLC, CCK-8 assay was performed. The data confirmed that anti-proliferative effect of hsa_circ_0018818 shRNA on NSCLC was further increased in the presence of AZD5363 (Figure 7A). Consistently, AZD5363 enhanced the apoptotic effect of hsa_circ_0018818 shRNA (Figure 7B). Moreover, the inhibitory effect of hsa_circ_0018818 shRNA on cell invasion was enhanced by AZD5363 as well (Figure 7C). To sum up, Akt inhibitor further enhanced the inhibitory effect of hsa_circ_0018818 shRNA on progression of NSCLC.

### Hsa_circ_0018818 knockdown significantly inhibits NSCLC tumor growth *in vivo*

**Figure 8:**
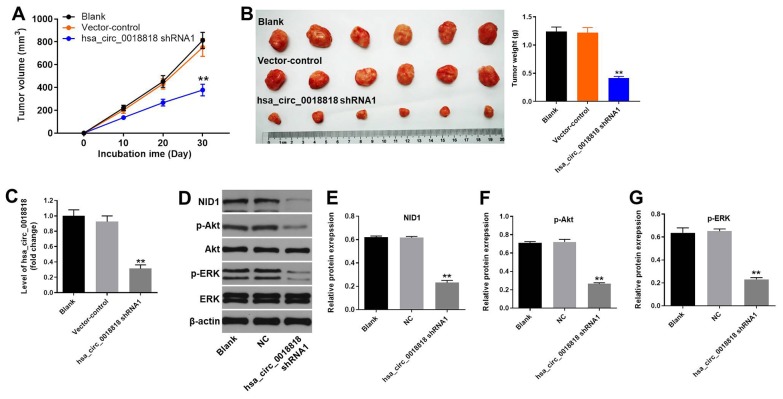
**Hsa_circ_0018818 shRNA1 suppresses NSCLC tumor growth *in vivo*.** Mice were subcutaneously injected NC1-H1650 transfected with vector-control or hsa_circ_0018818 shRNA1 or left untreated (Blank), after which tumors were allowed to grow for 4 weeks. (**A**) Volumes of tumors collected at the indicted times after transplantation. (**B**) Images of tumors (left) and tumor weights (right) after 4 weeks. (**C**) RT-qPCR analysis of hsa_circ_0018818 gene expression in tumor tissues. (**D**) Western blot analysis of NID1, Akt, ERK, p-Akt and p-ERK levels in tumor tissues. (**E**–**G**) Relative levels of NID1, p-Akt and p-ERK expression normalized to β-actin expression. **P< 0.01 vs. control.

Finally, a xenograft mouse model was established to detect the function of hsa_circ_0018818 in NSCLC *in vivo*. Four weeks after subcutaneous injection of NC1-H1650 cells, tumor size and weight were significantly lower when cells were transfected hsa_circ_0018818 shRNA1 prior to injection (Figure 8A, 8B). RT-qPCR confirmed that hsa_circ_0018818 levels were stably suppressed within the tumor tissue (Figure 8C). In addition, western blotting revealed that expression levels of NID1, p-Akt and p-ERK were all significantly reduced in tumor tissues expressing hsa_circ_0018818 shRNA1 (Figure 8D–8G). These results demonstrate that downregulating hsa_circ_0018818 significantly attenuates NSCLC tumorigenesis *in vivo*.

According to Union for International Cancer Control, version 8.

## DISCUSSION

CircRNAs, which are species of noncoding RNAs widely distributed in humans [12], different from linear noncoding RNAs such as miRNAs. It has been reported that circRNAs may mediate upregulation or downregulation of gene expression and, despite being classified as noncoding, may also encode proteins [13]. Moreover, circRNAs are stable and widely expressed in many tumor tissues [14, 15]. Some circRNAs have important biological functions and can be considered as biomarkers for diagnosis of multiple diseases [4, 16, 17]. This suggests the possibility that circRNAs, like miRNAs, may be involved in paracrine signaling or cell-to-cell crosstalk. Our findings indicate that hsa_circ_0018818 downregulation suppresses NSCLC cell proliferation and induces apoptosis, which is consistent with earlier reports indicating that circRNAs regulate the progression of NSCLC [9, 18, 19]. These findings suggest that hsa_circ_0018818 likely acts to promote tumorigenesis of NSCLC, particularly during the early stages of the disease. This may make hsa_circ_0018818 an important biomarker for diagnosis of early stage NSCLC.

MiRNAs are known to play important roles in the progression of multiple diseases, including NSCLC [20, 21]. We found that a miR-767-3p antagonist partially reversed the inhibitory effect of hsa_circ_0018818 knockdown on expression of NID1, p-Akt and p-ERK. Wan et al reported that miR-767-3p induces downregulation of lung adenocarcinoma cell proliferation and induce apoptosis [22]. Our findings suggest miR-767-3p is also a key regulator of NSCLC progression. In addition, Jiang et al demonstrated that hsa_circ_0000673 enhances hepatocellular carcinoma malignancy by sponging miR-767-3p [23]. These results are similar to our present findings, indicating that hsa_circ_0018818 knockdown suppresses the tumorigenesis of NSCLC by sponging miR-767-3p.

It was previously reported that NID1 plays a key role in multiple malignant tumors [24, 25]. For example, NID1 reportedly regulates such functions as cell proliferation, survival and metastasis [26, 27]. Our findings indicate that NID1 is a direct target of miR-767-3p. It was previously reported that miR-192 suppresses the progression of Hirschsprung's disease by directly targeting NID1 [28], and that NID1 acts as a tumor promoter in NSCLC [27]. These results further implicate NID1 in the development of NSCLC; indeed, the suggest NID1 may act as a key promoter in the occurrence of NSCLC. Otherwise, Jiang W et al indicated SET is the direct target of miR-767-3p in hepatocellular carcinoma [23]. This difference may due to the different tumor type.

Components of the PI3K/Akt pathway are targeted in more types of cancer than any other growth factor signaling pathway, and it is commonly activated as a cancer promoter [29]. It appears that the PI3K/Akt pathway is composed of multiple bifurcating and converging kinase cascades, supplying numerous potential targets for tumor therapy [30, 31]. In our current research, hsa_circ_0018818 knockdown significantly inactivated PI3K/Akt signaling. An earlier report similarly found that inactivation of PI3K/Akt signaling contributes to NSCLC cell apoptosis [32]. Moreover, our findings suggested that knockdown of hsa_circ_0018818 inactivated EMT in NSCLC cells. Ya Zhou et al revealed that NID1 could activate NID1/PI3K/Akt/EMT to regulate the tumorigenesis of ovarian cancer [33]. Together with that report, our results suggest NID1 promotes EMT process in NSCLC through activation of PI3K/Akt. Frankly speaking, this study only focused on the effect of hsa_circ_0018818 on PI3K/Akt signaling so far. Given that ASK1/JNK signaling reportedly acts as a tumor suppressor during the pathogenesis of NSCLC [34], we will investigate the effect of hsa_circ_0018818 on ASK1/JNK signaling in a future study.

In summary, we found that hsa_circ_0018818 was upregulated in NSCLC. Moreover, knockdown of hsa_circ_0018818 could inhibit NSCLC tumorigenesis by mediating miR-767-3p sponging and NID1/PI3K/Akt/EMT axis, making it a potential biomarker for the prognosis and treatment of NSCLC.

## MATERIALS AND METHODS

### Cell culture

The BEAS-2B, A549, PC-9, NCI-H441, NCI-H1650 and 293T cell lines were obtained from the American Type Culture Collection (ATCC, Manassas, VA, USA) and cultured in Dulbecco’s Modified Eagle’s Medium (DMEM, Thermo Fisher Scientific, Waltham, MA, USA) supplemented with 10% FBS (Thermo Fischer Scientific), 1% penicillin and streptomycin (Thermo Fisher Scientific) at 37°C under a 5% CO_2_ atmosphere. AZD5363 (Akt inhibitor) was obtained from MedChemExpress (MCE, Monmouth Junction, NJ, USA).

### Bioinformatics analysis

Three datasets (GSE101586, GSE101684 and GSE112214) containing the gene expression data for NSCLC and adjacent normal tissue (controls) were obtained from the GEO database (https://www.ncbi.nlm.nih.gov/geo/). Among them, GSE101684 was a dataset from a patient with early stage of NSCLC. Gene Ontology (GO) analysis was performed to explore the functional roles of circRNA-targeted genes in terms of biological processes, cellular components, and molecular functions. Biological pathways were defined using the Kyoto Encyclopedia of Genes and Genomes (KEGG).

### Tissue collection

In total, 30 pairs of NSCLC samples and adjacent normal tissues were collected from Peking Union Medical College Hospital between June 2018 and June 2019. The clinical and pathological data of these patients were collected with their written informed consent. Each tissue sample was stored at -80°C until RNA extraction. The present study was approved by the Ethics Committee of Peking Union Medical College Hospital. The correlation of hsa_circ_0018818 and clinic-pathological parameters of patients with NSCLC was presented in Table 1.

### Quantitative real time polymerase chain reaction (RT-qPCR)

Total RNA was extracted from NSCLC cell lines or tissues using TRIzol reagent (TaKaRa, Tokyo, Japan) according to the manufacturer's protocol. cDNA was synthesized using a reverse transcription kit (TaKaRa, Ver.3.0) according to the manufacturer's protocol. Real-Time qPCRs were performed in triplicate using the following protocol: 2 min at 94°C, followed by 35 cycles or 30 s at 94°C and 45 s at 55°C. The primers for hsa_circ_0018818, miR-767-3p, β-actin and U6 were obtained from GenePharma (Shanghai, China): for Hsa_circ_0018818, 5’-CCCACAGTTTTGCTCTGCAG-3’ (forward) and 5’-AGGTGTAGCCTGAGAAGTACGC-3’ (reverse); for MiR-767-3p, 5’-TCCATTTGTTTTGATGATGGACT-3’ (forward) and 5’-CTCAACTGGTGTCGTGGAGTC-3’ (reverse); for β-actin, 5’-GTCCACCGCAAATGCTTCTA-3’ (forward) and 5’-TGCTGTCACCTTCACCGTTC-3’ (reverse); and for U6, 5’-CTCGCTTCGGCAGCACAT-3’ (forward) and 5’-AACGCTTCACGAATTTGCGT-3’ (reverse). The relative fold changes were calculated using the 2^-ΔΔCt^ method with the formula 2-(sample ΔCt – control ΔCt), where ΔCt is the difference between the amplification fluorescent thresholds of the gene of interest and the internal reference gene (U6 or β-actin) used for normalization.

### Cell transfection

PcDNA3.1 expression vector encoding short-hairpin RNA (shRNA1 or shRNA2) targeting hsa_circ_0018818 or a non-targeted sequence (negative control) were obtained from GenScript Co., Ltd (Nanjing, China). NSCLC cells were then transfected with the vector using Liposome 2000, after which the transfectants were selected by incubation in the presence of G418 (Sigma Aldrich, St. Louis, MO, USA). RT-qPCR was used to verify the transfection efficiency. The shRNA sequences were as follows: hsa_circ_0018818 shRNA1, 5’-CTTCCCACAGTTTTGCTCTG-3’ (forward) and AAAACTTCCCACAGTTTTG (reverse); hsa_circ_0018818 shRNA2, 5’-CAGTTTTGCTCTGCAGACGG-3’ (forward) and AAAAACAGTTTTGCTCTGCA (reverse).

In addition, NCI-H1650 cells were transfected with miR-767-3p agonist, miR-767-3p antagonist or negative control (NC) RNAs (GenePharma, Shanghai, China) using Lipofectamine 2000 as previously described [35]. The transfection efficiency was determined using q-PCR.

### CCK-8 assays

A549 or NCI-H1650 cells were seeded into 96-well plates (5×10^3^ per well) and incubated overnight at 37°C. The cells were then treated with NC, hsa_circ_0018818 shRNA1 or hsa_circ_0018818 shRNA1 + AZD5363 (Akt inhibitor) for 0, 24, 48 or 72 h, after which 10 μl of CCK-8 reagent were added to each well, and the cells were incubated for an additional 2 h. Finally, the absorbance at 450 nm was measured using a microplate reader (Thermo Fisher Scientific).

### Cell apoptosis analysis

A549 or NCI-H1650 cells were trypsinized, washed with phosphate-buffered saline, resuspended in Annexin V Binding Buffer, and stained with 5 μl of FITC and 5 μl of propidium (PI) for 15 min. The cells were the analyzed using a flow cytometer (BD, Franklin Lake, NJ, USA) to assess the incidence of cell apoptosis.

### Transwell assays

The invasiveness of the cells was assessed using transwell assays. The upper chamber is pre-treated with 100 μl of Matrigel, after which A549 or NCI-H1650 cells were seeded into the upper chamber in RPMI1640 medium with 1% FBS. The density was adjusted to about 1.0×10^6^ cells per chamber. RPMI1640 medium with 10% FBS was added in the lower chamber. After incubation for 48 h at 37°C, the non-invading cells in the upper chamber were removed with a cotton swab. Cells in the lower chamber were stained with 0.1% crystal violet and counted under a microscope (LEICADMLB2, Frankfurt, Germany).

### Dual luciferase reporter assay

The partial sequences of hsa_circ_0018818 and the NID1 3’-UTR containing putative miR-767-3p binding sites were obtained from Sangon Biotech (Shanghai, China) and cloned into pmirGLO Dual-Luciferase miRNA Target Expression Vectors (Promega, Madison, WI, USA) to construct wild-type reporter vectors hsa_circ_0018818 (WT) and NID1 (WT), respectively. Mutant hsa_circ_0018818 and NID1 3’-UTR sequences containing the putative miR-767-3p binding site were generated using a Q5 Site-Directed Mutagenesis Kit (New England Biolabs, Ipswich, MA, USA) and then cloned into pmirGLO vectors to generate the mutant-type reporter vectors hsa_circ_0018818 (MUT) and NID1 (MUT). hsa_circ_0018818 (WT) or hsa_circ_0018818 (MUT) were transfected into 293T cells together with NC or miR-767-3p agonist using Lipofectamine 2000 (Thermo Fisher Scientific). Similarly, NID1 (WT) or NID1 (MUT) was transfected into 293T cells together with NC or miR-767-3p agonist. Relative luciferase activities were then analyzed using a Dual-Glo Luciferase Assay System (Promega).

### Fluorescence in situ hybridization (FISH)

To explore the relation between hsa_circ_0018818 and miR-767-3p, their colocalization in the cytoplasm was investigated using FISH as previously described [36].

### Western blotting

Total protein was isolated from cell lysates or tumor tissues using RIPA buffer, and quantified using a BCA protein assay kit (Beyotime, Shanghai, China). Proteins were resolved on 10% SDS-PAGE and then transferred to PVDF (Bio-Rad) membranes. After blocking, the membranes were incubated first with primary antibodies at 4°C overnight and then with secondary anti-rabbit antibody (Abcam; 1:5000) at room temperature for 1 h. Membranes were scanned using an Odyssey Imaging System and analyzed using Odyssey v2.0 software (LICOR Biosciences, Lincoln, NE, USA). The primary antibodies were anti-Akt (Abcam, Cambridge, MA, USA; 1:1000), anti-ERK (Abcam; 1:1000), anti-NID1 (Abcam; 1:1000), anti-E-cadherin (Abcam; 1:1000), anti-Twist2 (Abcam; 1:1000), anti-Vimentin (Abcam; 1:1000) and anti-β-actin (Abcam; 1:1000). β-actin served as an internal control.

### *In vivo* study

Eighteen BALB/c nude mice (6-8 weeks old) were purchased from Vital River (Beijing, China) and housed within a dedicated SPF facility. NCI-H1650 cells stably expressing hsa_circ_0018818 shRNA1 were transplanted subcutaneously into each mouse as described previously [37]. Tumor volume was then measured weekly as described previously [38]. At the end of the experiment, the mice were sacrificed and the tumors were collected and weighted. All *in vivo* experiments were performed in accordance with National Institutes of Health Guide for the Care and Use of Laboratory Animals. The protocol was approved by the Ethics Committees of Peking Union Medical College Hospital.

### Statistical analysis

All data were expressed as the mean ± standard deviation (SD) of at least three independent experiments. Differences were analyzed using one-way analysis of variance (ANOVA) followed by Tukey’s test (more than 2 groups, Graphpad Prism7). Values of P<0.05 was considered statistically significant.
